# Safety, Tolerability, and Immunogenicity of a DNA Vaccine (pGX9501) Against SARS-CoV-2 in Healthy Volunteers: A Single-Center, Randomized, Double-Blind, Placebo-Controlled, and Dose-Ranging Phase I Trial

**DOI:** 10.3390/vaccines13060573

**Published:** 2025-05-27

**Authors:** Haijing Yang, Yang Zhou, Xin Cheng, Chao Qiu, Shuo Wang, Yu Xia, Xuefen Huai, Zhenning Xiu, Jiarong Wang, Yue He, Guoying Cao, Qiong Wei, Jingjing Wang, Jingwen Ai, Haochen Zhang, Yi Zhang, Jing Zhang, Wenhong Zhang, Bin Wang

**Affiliations:** 1 Phase I Clinical Research Center, Huashan Hospital, Fudan University, Shanghai 200400, China; 2 Department of Infectious Disease, Huashan Hospital Affiliated to Fudan University, Shanghai 200400, China; 3 Advaccine Biopharmaceuticals Suzhou Co., Ltd., Suzhou 215000, China; 4 Key Laboratory of Medical Molecular Virology (MOE/NHC/CAMS), School of Basic Medical Science, Fudan University, Shanghai 200032, China

**Keywords:** COVID-19, SARS-CoV-2, DNA vaccine, pGX9501(INO-4800), acute respiratory disease

## Abstract

**Background**: pGX9501 is a prophylactic DNA vaccine encoding the spike protein of SARS-CoV-2 and can induce immune response in the human body so as to prevent COVID-19. With respect to non-clinical studies, pGX9501 has been demonstrated to induce both cellular and humoral immune responses in various animal models. It was found that the level of antibody titers following a two-dose regimen was higher than that following a single-dose regimen in nonhuman primate challenge model. **Methods**: In China, a phase I, randomized, double-blind, placebo-controlled clinical trial has been conducted in Huashan Hospital, Shanghai, China to evaluate the safety, tolerability, and immunogenicity of DNA vaccine pGX9501 administered intradermally (ID) followed by electroporation (EP) in 45 Chinese healthy volunteers aged 18 to 59 years old. **Results**: No adverse events of special interest (AESIs), death, or treatment-related SAEs occurred in this study. All the treatment-related (vaccine or EP) adverse events (TRAEs) were of grade 1 and 2 in severity. The solicited AEs were reported in thirty-two (32/36, 88.9%) and nine (9/9, 100.0%) subjects, respectively, in the DNA vaccine and placebo group. The frequency of solicited AEs did not increase with vaccine dose level and frequency. The DNA vaccine pGX9501 effectively enhanced both humoral and cellular immune responses in a dose-dependent manner, with increased antibody GMTs and peak seroconversion rates observed on day 42. The significant rise in IFN-γ levels confirmed the vaccine’s ability to induce cellular immune responses. Variations in the microbiome structure suggested a tangible impact of the gut microbiota on vaccine immunogenicity. **Conclusions**: The findings from this study confirm the immunogenicity and safety of the DNA vaccine pGX9501 and point to the potential role of the gut microbiota in vaccine immune responses. These insights provide practical references for the future design and development of DNA vaccines.

## 1. Introduction

A novel approach using synthetic DNA-based vaccine technology to vaccinate against a broad array of target antigens and diseases has been in development over the past 20 years. Compared with other types of vaccines, DNA vaccines have several advantages, such as the ability of stimulating balanced B-and T-cell responses and being well tolerated [1,2], stability of the vaccine across a broad temperature range, absence of infectivity of the immunogen itself, rapid construction of DNA plasmids with targeted antigen sequences, and a straightforward and scalable manufacturing process. Based on the DNA vaccine pathway, plasmid DNA can be more effective in producing the proteins encoded by the DNA expression plasmid system after it is ingested by the cell.

The integration of electroporation (EP) technology further enhances the efficient delivery of DNA vaccines into host cells, thereby improving cellular uptake and immunogenicity [3].

pGX9501 is a DNA vaccine encoding the SARS-CoV-2 spike protein, which had demonstrated promising protective potential and favorable safety profile based on available information from comprehensive preclinical studies [4]. In the initial phase I clinical trial of pGX9501 (NCT04336410) conducted in the US, 39 out of 40 participants (97.5%) successfully received both doses of the DNA vaccine. No adverse events of special interest (AESIs) or serious adverse events (SAEs) were observed. The study demonstrated robust immune response, with all 38 evaluable participants exhibiting either antibody or T-cell-mediated immunity after two doses. Specifically, 95% (18/19) of subjects in each dosage cohort showed measurable humoral responses, as assessed through binding or neutralizing antibody assays. Neutralizing antibodies, detected via live virus neutralization testing, were present in 78% (14/18) and 84% (16/19) of participants in the 1.0 mg and 2.0 mg dose groups, respectively. Geometric mean titers (GMTs) were 102.3 [95% CI (37.4, 280.3)] for the 1 mg dose and 63.5 [95% CI (39.6, 101.8)] for the 2 mg dose [5]. Additional supports for pGX9501’s clinical potential come from similar DNA vaccine trials, such as INO-4700, a MERS-CoV (Middle East respiratory syndrome coronavirus) DNA vaccine with a comparable design [6,7]. Our study was a phase I, single-site, randomized, double-blind, placebo-controlled investigation evaluating three different dosage levels of pGX9501 in 45 healthy Chinese adults. The vaccine was delivered via intradermal injection followed by electroporation using the CELLECTRA^®^ 2000 system (Inovio Pharmaceuticals, Inc., San Diego, CA, USA). This report summarizes the safety, tolerability, and immunogenicity findings from this trial.

## 2. Methods

### 2.1. Study Design and Participants

This phase I trial of the DNA vaccine pGX9501 was done at the Phase I Unit, Huashan Hospital, Fudan University, Shanghai, China (ChiCTR2000038152). Written informed consent from each participant was obtained before screening. This was a single-center, randomized, double-blinded, placebo-controlled, dose-ranging phase I study to evaluate the safety, tolerability, and immunogenicity of INO-4800 administered intradermally followed by electroporation in healthy volunteers. During the trial, there were three stages, including the screening period (14 days prior to dosing), immunization period (vaccination on day 0 and day 28), and follow-up period (from day 29 to day 365, or dropout). A total of 45 healthy volunteers who met the inclusion and exclusion criteria were enrolled after at most 14 days of screening and evaluated across 3 dose levels (low dose, at 0.5 mg; medium dose, at 1.0 mg, and high dose, at 2.0 mg), each of which included 15 participants (an initial 3 of 15 as sentinels having the vaccine administrated, 9 of 15 as participants having the vaccine administrated, and 3 of 15 having a placebo administrated). The 45 healthy participants were aged 18~59 years with a body mass index of 18~30 kg/m^2^ and had tested negative for SARS-CoV-2 exposure, as confirmed by laboratory tests via a RT-PCR assay or chest CT image. The kit for SARS-CoV-2 detection was from Daan Gene Co., Ltd. (DA0992, DA AN gene, Guangzhou, China), in which the SARS-CoV-2 ORF1ab and N genes were detected as the genomic targets. Vital signs, physical examination, and electrocardiogram (ECG) results within normal range or abnormal range with no clinically significant meanings were screened for enrollment. Exclusion criteria included positive serum pregnancy test during screening; positive serological tests for hepatitis B surface antigen (HBsAg), hepatitis C antibody, syphilis, and Human Immunodeficiency Virus (HIV) infection; hypersensitivity or severe allergic reactions to any ingredient in the vaccine; or having a history of mental illness. Further details are summarized in the trial protocol.

### 2.2. Declaration

This study was undertaken in accordance with the principles of the Declaration of Helsinki and Good Clinical Practice. Ethic approval was obtained from Shanghai Ethics Committee for Clinical Research (No. SECCR/2020-20-02).

### 2.3. Randomization and Masking

The participants were sequentially allocated to one of three cohorts (0.5 mg, 1.0 mg, and 2.0 mg dose level) to receive the DNA vaccine pGX9501 or a placebo at day 0 and day 28 by intradermal injection followed with electroporation. There were 15 participants in each dose cohort, including 3 sentinels receiving pGX9501, and the remaining 12 subjects were randomly assigned with a ratio of 9:3 to receive pGX9501 or a placebo. A double-blind method was employed for this trial. The clinical investigators and the participants were kept blind to the group allocation throughout the trial.

### 2.4. Objectives and Endpoints

The primary objective was to evaluate the safety of pGX9501. The primary endpoint was the frequency of adverse events within 28 days after the 1st dose and 30 days after the 2nd vaccination by severity and relationship with pGX9501. The secondary objective was to assess immunogenicity, especially the humoral immune response. Key immunogenicity endpoints in this phase I trial included antigen-specific binding antibody response, neutralization antibody response at day 0 and days 42 and 58 after the 1st vaccination. The cellular response and gut microbiota composition data were also explored in this trial.

### 2.5. Procedures

The active investigational product, which contains 10 mg/mL of pGX9501 expressing a synthetic consensus (SynCon^®^, Inovio Pharmaceuticals, Inc., San Diego, CA, USA). sequence of the SARS-CoV-2 full length spike protein in 1xSSC buffer (150 mM sodium chloride and 15 mM sodium citrate), was supplied in glass vial. The corresponding placebo contains the vaccine excipients except the DNA plasmid pGX9501. This study consisted of three stages: a screening period, vaccination period, and follow-up period. There were three cohorts planned, with 15 participants in each group. Starting from the three sentinels of the low-dose group (0.5 mg), pGX9501 was administered intradermally, followed by electroporation between each sentinel at a 1 h interval on day 0, and safety data were observed and recorded within the 7 days following the 1st vaccination of each cohort’s 3 sentinels. Afterwards, the data safety monitoring board (DSMB) would review and evaluate all available safety and tolerability results related to the sentinels and issue a decision on the enrollment of the 12 remaining participants for each cohort, as well as the dose escalation to the next dose cohort. Those who did not trigger the contraindications described in the protocol would receive the second dose on day 28 and then enter the follow-up stage, which lasted until the end of study.

#### 2.5.1. Adverse Events Collection

An independent data safety monitoring board (DSMB) reviewed the safety data at week 1 after the first dose was initiated and made a recommendation on whether to complete enrollment of the additional 12 subjects into that dose group and, additionally, the 3 sentinel subjects into the next higher dose group. Enrollment into the next higher dose group subsequently occurred in a similar fashion. Subjects received a 24 h inpatient care after each dose to avoid any emergent safety concerns, like acute hypersensitive reaction. Follow-up visits were scheduled after each dose until day 365 (end of study). Solicited injection-site and systemic AEs were recorded within 14 days after each dose and unsolicited AEs were recorded in diary cards for about 28 days after each dose. Injection-site reactions, systemic symptoms, SAEs, AESIs, and unexpected adverse events were monitored throughout the study. All the AEs, regardless of relationship to the vaccine or EP, were assessed, graded, and recorded by an investigator. The severity of AEs was determined according to the Common Terminology Criteria for Adverse Events (CTCAE) version 5.0 and the guidelines on the “Classification Standards for Adverse Events in Clinical Trials of Preventive Vaccines” released by NMPA on 31 December 2019 [8,9].

During this phase I clinical trial, all adverse events, including solicited and unsolicited adverse events occurring 0~28 days after the 1st vaccination and within 30 days after the 2nd vaccination, were recorded by participants on diary cards and verified by investigators. Adverse events and laboratory abnormalities were graded in accordance with Common Terminology Criteria for Adverse Events (CTCAE 5.0) and the Guidelines for Grading Criteria for Adverse Events in Clinical Trials of Prophylactic Vaccines issued by the National Medical Products Administration of China (NMPA).

#### 2.5.2. Immunogenicity Analysis

Blood samples from participants at designated time points were collected by site staff to assess both humoral and cellular immune responses, including the detection of binding and neutralizing antibodies, as well as IFN-γ responses.

The binding antibody assay employed the ELISA method to detect SARS-CoV-2 spike-specific IgG antibody titers in human serum. Developed and validated by JOINN Laboratories, the assay was transferred to NIFDC for sample analysis. The procedure involved adding test serum samples to a plate pre-coated with SARS-CoV-2 spike protein (Acro Biosystems Group, Newark, DE, USA. Cat No.: SPN-C52H9, Batch No.: C591P1-20B2F1-UQ), followed by the addition of HRP-labeled anti-human IgG secondary antibodies (BD Biosciences Pharmingen, San Diego, CA, USA. Cat No.: 555788, Batch No.: 1188970) to form a complex. A substrate was used to induce color development and the reaction was stopped with concentrated sulfuric acid. Absorbance at 450 nm was measured, with signal strength correlating to the amount of anti-SARS-CoV-2 spike IgG antibodies. A ratio of OD values ≥ 2.1 between post- and pre-immunization samples at the same dilution level indicated a positive result, with the highest dilution level representing the antibody titer. Quality control was ensured with positive control (PC) samples and normal negative serum samples. The positive control antibody was a Human Anti-SARS-CoV-2 S1 Monoclonal Antibody (Creative Biolabs, Shirley, NY, USA. Cat No.: CBMAB-R0120-FY, Batch No.: CB2108LY03).

The neutralizing antibody assay was performed in the BSL-3 laboratory of the Jiangsu Provincial Center for Disease Control and Prevention in China. The test virus strain, isolated from clinical COVID-19 cases, was sequenced and identified as the Beta variant (National Collection Unique Number: CSTR.16698.06. NPRC 2.062100001). A microneutralization assay based on the cytopathic effect (CPE) was used to evaluate the neutralizing antibody activity. Fixed volumes of the virus were mixed with sample dilutions and applied to infect Vero-E6 cell monolayers, with each dilution tested in duplicate. The experiment incorporated normal cell controls, negative and positive antibody controls, and a back-titration for virus titration quality control. On the third day post-inoculation, the presence or absence of CPE in the cells was assessed. If the negative control cells maintained normal morphology and the virus control, set at 100 TCID_50_ per well, fell within the range of 32-320 TCID_50_, the test wells were scored. The neutralizing titer was determined as the highest serum dilution that could protect 50% of the cell wells from viral infection.

The IFN-γ assay was conducted utilizing a commercial ELISpot kit (Mabtech, Inc., Cincinnati, OH, USA. Cat No.: 3420-2APW-10) by JOINN Laboratories (Beijing, China). Peripheral blood mononuclear cells (PBMCs) were isolated from pre- and post-vaccinated individuals’ K_2_EDTA anticoagulated peripheral blood and stored in liquid nitrogen for batch analysis. Following a recovery process involving overnight incubation at 37 °C with 5% CO_2_, the cells were stimulated with five peptide pools derived from the full-length spike protein of the SARS-CoV-2 (Pool 1: amino acid sequences 1–268, Pool 2: sequences 260–520, Pool 3: sequences 512–772, Pool 4: sequences 764–1024, and Pool 5: sequences 1016–1273). Each cell sample was incubated with the respective peptide pools overnight. A phosphoacylase-labeled anti-IFN-γ antibody was employed as the detection reagent. After a series of washing steps, the results were visualized as spots, with each spot representing an individual IFN-γ-secreting cell. The plates were analyzed using an ELISpot Analyzer (AID GmbH, Strassberg, DE. QC-026-MR01). Each sample was assayed in triplicate. Negative controls (3 × 10^6^ cells/well with 1% DMSO) and positive controls (1.1 × 10^5^ cells/well with PMA and ionomycin) were included for each sample, also in triplicate. The test results were normalized to spot-forming units (SFUs) per 1 × 10^6^ cells.

#### 2.5.3. Gut Microbiota Composition Testing Method

An exploratory study was conducted to compare the relationship between microbiome profiles and immune responses to the COVID-19 DNA vaccines (Supplementary Table S1). A total of 36 stool samples were sequenced, yielding an average of 7.7 Gb (33.7 million reads) per sample. Participants were categorized based on their immune responses at two and four weeks post-completion of the full vaccination course.

Qualified data underwent a stringent quality control and filtering process prior to de novo assembly. Following this, gene prediction was performed on the assembled results. Clustering of the predicted genes yielded a non-redundant gene set. Annotation of these gene sets was conducted by comparing them with the humanGut_11M database [10] for gene function and species annotation. Reads were mapped back to the gene set, enabling the calculation of gene and species abundance for each sample. Species diversity analysis, cluster analysis, differential analysis, functional enrichment analysis, and other relevant analytical procedures were carried out using the gene and species abundance data.

### 2.6. Statistical Analysis

No statistical hypothesis was formulated in this study; instead, descriptive statistics were utilized to analyze the safety data as the primary outcome. GraphPad Prism 9 version 9.0.0 was employed to plot immunogenicity figures. To discern the geometric mean titer (GMT) differences in binding and neutralizing antibody responses among cohorts, Wilcoxon tests were conducted. All statistical analyses were two-tailed, and a *p*-value of less than 0.05 was set as the threshold for statistical significance.

## 3. Results

### 3.1. Study Population

**Figure 1:**
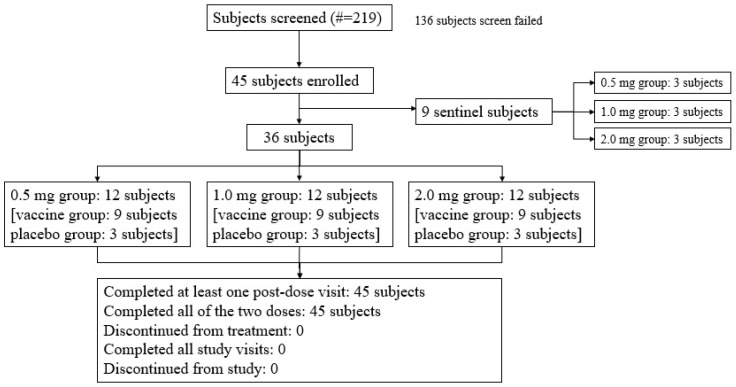
Subject screening and disposition (all subjects). A total of 45 subjects were enrolled, including 9 sentinel subjects across the low-dose 0.5 mg, medium-dose 1.0 mg, and high-dose 2.0 mg vaccine groups, each of whom received the investigational vaccine. In addition to these 9 sentinel subjects, 36 subjects, which constituted 80.0% of the enrolled subjects, were randomized into the vaccine and placebo groups in a 9:3 ratio.

Three sentinel subjects in each dose group (the low-dose 0.5 mg vaccine group, medium-dose 1.0 mg vaccine group, and high-dose 2.0 mg vaccine group) received the investigated vaccine. Among the enrolled subjects, except for the nine sentinel subjects, thirty-six subjects (80.0%) were randomized to be divided into the vaccine group or the placebo group at a ratio of 9:3 (Figure 1).

### 3.2. Demographic and Baseline Characteristics

The demographics and baseline characteristics were generally balanced and comparable across all cohorts (Table 1). Among the 45 enrolled subjects, there were 31 male subjects (68.9%) and 14 female subjects (31.1%). The mean age was 30.5 ± 9.57 years, with a range of 20–54 years, and 38 subjects (84.4%) were 40 years old or younger. All 45 enrolled subjects were Asian, of which 42 subjects (93.3%) were of Han Chinese ethnicity.

### 3.3. Safety and Tolerability

In total, forty-three subjects completed the study and two subjects reported an early withdrawal. One subject in the 0.5 mg group withdrew on day 278 due to pregnancy, and another subject in the 1.0 mg group withdrew on day 309 for personal reasons, specifically military service. No subject discontinued the trial due to an adverse event (AE). A subject who received two doses of the 2 mg DNA vaccine reported a serious adverse event (SAE) on day 304 due to tibia and fibula fractures. This subject was treated with medication, brace fixation, open reduction, and internal fixation and recovered approximately 70 days later. An investigator determined that this event was not related to the DNA vaccine. No adverse events of special interest (AESIs), deaths, or drug-related SAEs occurred in this study.

**Figure 2:**
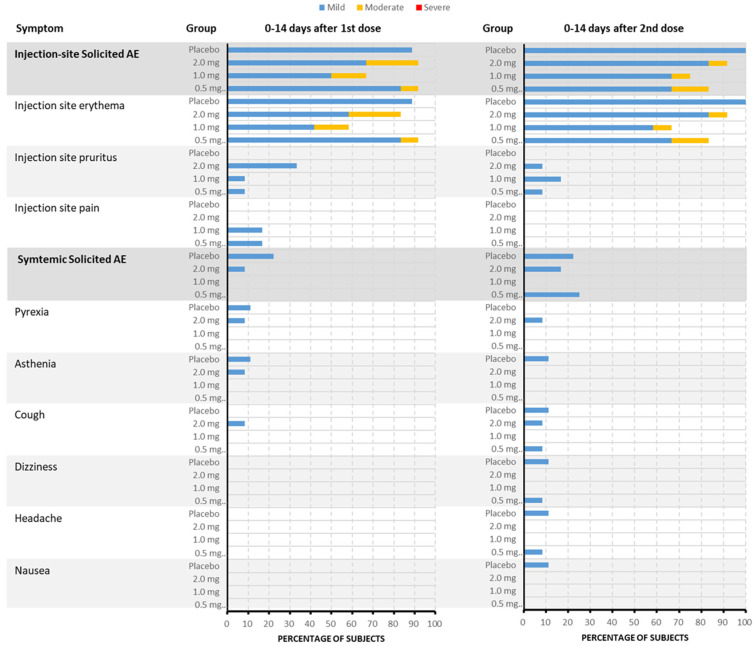
The incidence and severity of solicited TEAEs after each dose in each group (by PT). Within the vaccine group, the most frequently reported injection-site adverse events were erythema at the injection site (83.3%), pruritus (22.2%), and pain (11.1%). Among the 45 subjects included in the Safety Set, about 41 subjects (91.1%) experienced solicited TEAEs. When judged by the most severe grade, about 34 subjects (75.6%) experienced grade 1 events and 7 other subjects (15.6%) experienced grade 2 events. The incidence of solicited TEAEs in the 1.0 mg vaccine group was 83.3%, which was lower than that in the 0.5 mg vaccine group, 2.0 mg vaccine group, and placebo group, in which the incidence of solicited TEAEs was 91.7%, 91.7%, and 100.0%, respectively. Additionally, systemic solicited adverse events were reported in 25.0% of subjects in the 0.5 mg group, 0% in the 1.0 mg group, 16.7% in the 2.0 mg group, and 33.3% in the placebo group. The systemic adverse events primarily included fever (5.6%), cough (5.6%), asthenia (2.8%), dizziness (2.8%), and headache (2.8%) within the vaccine group.

Treatment-emergent adverse events (TEAEs) were reported in 43 subjects (43/45, 95.6%), including thirty-four subjects (34/36, 94.4%) who received the DNA vaccine and nine subjects (9/9, 100%) who received the placebo. All TEAEs, except for the aforementioned SAE, were of grade 1 or 2. Treatment-related laboratory AEs were all of grade 1. The incidence rate of solicited AEs in the 0.5 mg, 1.0 mg, 2.0 mg, and placebo groups was 91.7% (11/12), 83.3% (10/12), 91.7% (11/12), and 100.0% (9/9), respectively. All solicited AEs were judged to be related to treatment, except for one case of “headache.” The rate of solicited injection-site AEs was consistent with the solicited AEs in the three vaccine groups and the placebo group. The most frequent solicited injection-site AEs in the vaccine group were injection-site erythema (83.3%), pruritus (22.2%), and pain (11.1%). Systemic solicited AEs were reported in three (25.0%), zero, two (16.7%), and three (33.3%) subjects in the 0.5 mg, 1.0 mg, 2.0 mg, and placebo groups, respectively, mainly including fever (5.6%), cough (5.6%), asthenia (2.8%), dizziness (2.8%), and headache (2.8%) in the vaccine group (Figure 2). The incidence of unsolicited AEs in the vaccine and placebo groups was 80.6% (29/36) and 66.7% (6/9), respectively. Thirteen subjects (36.1%), all judged to be related to treatment, were in the vaccine groups.

Compared with the incidence rate of solicited AEs after the first dose, there was no increase in the frequency of solicited AEs after the second dose, both being 83.3% (30/36).

### 3.4. Immunogenicity

#### 3.4.1. Spike-Binding Antibody

**Figure 3:**
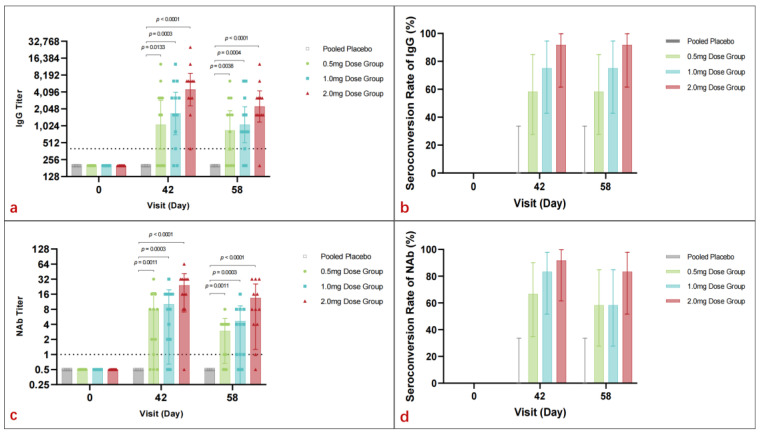
The humoral response of pGX9501 in each group. (**a**) Geometric mean titers (GMT) of binding antibodies to pGX9501 on day 42 demonstrated a significant positive correlation with vaccine dosage, with peaks at 1067.9 for the 0.5 mg group, 1695.1 for the 1.0 mg group, and 4525.5 for the 2.0 mg group. These elevated GMT values were largely maintained by day 58. (**b**) Seroconversion rates of binding antibodies peaked at 58.3% for the 0.5 mg group, 75.0% for the 1.0 mg group, and 91.7% for the 2.0 mg group on day 42 and remained consistent by day 58. (**c**) GMT of neutralizing antibodies. All participants in the placebo group tested negative throughout the study, while vaccine-induced GMTs increased with dosage, reaching a peak in the 2.0 mg group on day 42 and sustained through day 58. (**d**) Seroconversion rates of neutralizing antibodies. Regarding the antibody titers, the 2.0 mg group exhibited the highest rates, demonstrating a correlation between the immune response and vaccine dosage.

On day 42, vaccinated participants demonstrated significant geometric mean titer (GMT) peaks for binding antibodies: 1067.9 (95% CI: 392.1–2908.0) for the 0.5 mg group, 1695.1 (95% CI: 710.3–4045.7) for the 1.0 mg group, and 4525.5 (95% CI: 2329.8–8790.4) for the 2.0 mg group. A positive correlation between dosage and GMT was evident. By day 58, GMT values remained high across vaccine groups: 847.6 (95% CI: 378.3–1899.2) for 0.5 mg, 1067.9 (95% CI: 510.3–2234.5) for 1.0 mg, and 2262.7 (95% CI: 1196.9–4277.6) for 2.0 mg, while the placebo group exhibited no significant GMT changes from baseline (Figure 3a).

Seroconversion rates for binding antibodies peaked on day 42 in all vaccine groups: 58.3% (95% CI: 27.7–84.8) for 0.5 mg, 75.0% (95% CI: 42.8–94.5) for 1.0 mg, and 91.7% (95% CI: 61.5–99.8) for 2.0 mg. Rates on day 58 were consistent with those on day 42 (Figure 3b).

#### 3.4.2. Neutralizing Antibody

All participants from the placebo cohort tested negative for neutralizing antibodies (Nab) before vaccination and throughout the study. On day 42, vaccine-induced Nab geometric mean titers (GMTs) were observed to be 3.4 (95% CI: 1.3–8.8) in the 0.5 mg group, 5.3 (95% CI: 2.2–13) in the 1.0 mg group, and 16.0 (95% CI: 7.2–35.5) in the 2.0 mg group. By day 58, the GMTs were 2.0 (95% CI: 1.0–3.8) for the 0.5 mg group, 2.7 (95% CI: 1.2–5.7) for the 1.0 mg group, and 7.6 (95% CI: 3.2–17.7) for the 2.0 mg group. Nab levels increased with dosage, peaking in the 2.0 mg group and being sustained through day 58 (Figure 3c).

Seroconversion rates on day 42 were 66.7% (95% CI: 34.9–90.1%) for the 0.5 mg group, 83.3% (95% CI: 51.6–97.9%) for the 1.0 mg group, and 91.7% (95% CI: 61.5–99.8%) for the 2.0 mg group. On day 58, the seroconversion rates were 58.3% (95% CI: 27.7–84.8%) for the 0.5 mg group, 58.3% (95% CI: 27.7–84.8%) for the 1.0 mg group, and 83.3% (95% CI: 51.6–97.9%) for the 2.0 mg group. Seroconversion rates increased with dosage, with the 2.0 mg group maintaining the highest rate, indicating a correlation between vaccine dosage and immune response (Figure 3d).

#### 3.4.3. IFN-γ Elispot Assay

**Figure 4:**
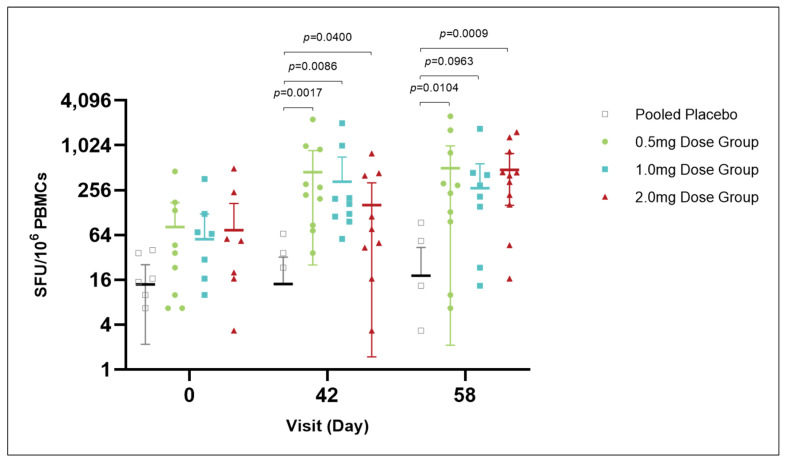
IFN-γ response over time in vaccine and placebo groups. At day 0, prior to vaccinate, IFN-γ levels were similar among all groups, with no significant differences observed. Post-vaccination on day 42, there was a notable increase in IFN-γ levels in the vaccinated groups compared to the placebo group, with the 0.5 mg, 1.0 mg, and 2.0 mg dose groups showing significant elevations. By day 58, these elevated IFN-γ levels were sustained, indicating a persistent activation of cell-mediated immune responses induced by vaccination. The placebo group’s IFN-γ levels remained unchanged, further highlighting the vaccine’s impact on immune activation.

At day 0, prior to vaccination, IFN-γ levels (SFU per 10^6^ PBMCs) were comparable across all groups, with the placebo group averaging 13.89 (95% CI: 2.18, 25.6) and the 0.5 mg, 1.0 mg, and 2.0 mg dose groups averaging 81.67 (95% CI: −10.63 to 173.96), 56.39 (95% CI: −9.22 to 121.99), and 74.17 (95% CI: −21.3 to 169.63), respectively, showing no significant differences. On day 42, vaccinated groups exhibited a significant increase in IFN-γ levels, with the 0.5 mg, 1.0 mg, and 2.0 mg dose groups averaging 448.06 (95% CI: 25.45 to 870.66), 332.79 (95% CI: −48.08 to 713.63), and 161.11 (95% CI: 1.48 to 320.74), respectively, significantly differing from the placebo group’s average of 14.7 (95% CI: −4.26 to 32.41). By day 58, IFN-γ levels remained elevated in the dose groups, with averages of 504.17 (95% CI: 2.12 to 1006.2), 270.83 (95% CI: −35.2 to 576.86), and 479.17 (95% CI: 160.76 to 797.57), respectively, still significantly different from the placebo group’s average of 18.15 (95% CI: −7.32 to 43.61). These results suggest that the vaccination induced sustained high levels of IFN-γ across the dose groups, activating cell-mediated immune responses (Figure 4).

### 3.5. Gut Microbiota Composition

**Figure 5:**
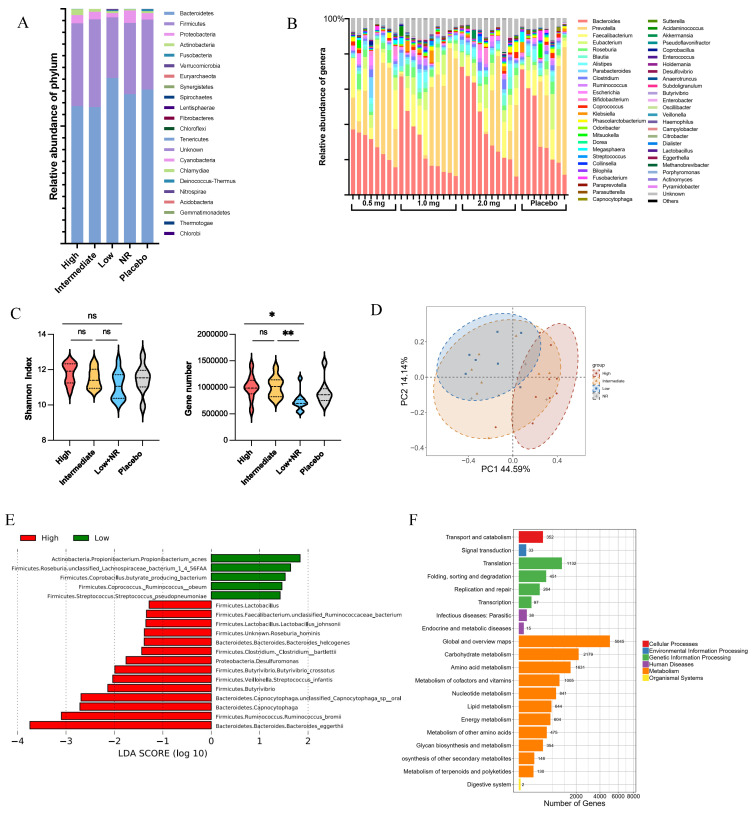
Differences in microbiome structure, beta diversity, alpha diversity, and bacterial species from baseline of vaccination. (**A**) Bacteroidetes and Firmicutes were dominant phylum in health vaccinee’s gut microbiome. (**B**) Composition of microbiome on genus level indicated individual differences in gut microbiota. (**C**) Alpha diversity varied between different vaccine response groups at baseline. The values were considered as follows: ns, non-significant. *, *p* < 0.05, **, *p* < 0.01 (**D**) Beta diversity at genus level was significantly different between high-response and low-response group at baseline (high, n = 9; low, n = 7). Principal component analysis (PCA) ordinations were used to visualize clustering of patients based on their species-level compositional profiles. (**E**) Differentially abundant species between high- and low-response groups detected using LDA Effect Size (LEfSe) analysis. (**F**) Functional pathway enriched in high-response vaccinee’s gut microbiome.

Shotgun metagenomic analysis was conducted on stool samples to investigate the relationship between baseline gut microbiome composition and the immune response to the COVID-19 DNA vaccines. DNA-vaccinated participants were divided into four response groups according to their antibody levels on study days 42 and 58, while the group that received the placebo still served as the control group (Supplementary Table S4). The gut microbiota of participants exhibited a structure typical of healthy humans, primarily dominated by Bacteroidetes (62.7 ± 17.7%) and Firmicutes (32.3 ± 16.5%) (Figure 5A). However, substantial individual variations were observed in the gut microbiome composition at the baseline (Figure 5B, Supplementary Figures S1 and S2A).

At the genus level, participants were categorized into three enterotypes based on the most abundant genus: Bacteroides Enterotypes, Prevotella Enterotypes, and Mixed Enterotypes, regardless of the vaccination dosage or their vaccine responses (Supplementary Figure S2B,C). This finding suggests a diversified microbiota in healthy participants. No significant differences were observed in the predominant microbial phylum and genus in the gut microbiome. However, there was a notable variation in the gut microbiome composition at the species and gene level.

The high-response group exhibited higher microbial diversity, including significantly more observed microbial genes and a relatively higher Shannon diversity index compared to the lower-response groups (Figure 5C). At the species level, variations in beta diversity between the high-response and low-response groups were observed (Figure 5D), with increased abundance of Bacteroides eggerthii, Ruminococcus bromii, and Streptococcus infantis in the high-response groups (Figure 5E). These differences in microbial taxa and genes between high- and low-response groups led to varied microbial functions, including higher metabolism in carbohydrates, amino acids, and lipids in the high-response group.

## 4. Discussion

On the nucleic vaccines platform, DNA vaccines show unique advantages over conventional vaccines, including stability, simplicity of vaccine production and transportation, and their capability to elicit balanced humoral and cellular immune responses [11,12,13]. Therefore, DNA vaccines have drawn increasing attention from scientists and the industry for a long time. Currently, there are more than ten DNA vaccines undergoing clinical trials worldwide and one India-developed DNA vaccine (ZyCoV-D) approved under emergency use authorization, which is a milestone in the history of DNA vaccine development and further promotes the development of DNA vaccines. Our DNA vaccine, pGX9501, can be stored for more than four years at a temperature of 2~8 °C and at least one year at room temperature, greatly improving a competitive stability profile among vaccine products. As a result, there is a great prospect for DNA vaccines in the future.

In addition, the safety profile of DNA vaccines had been demonstrated by variety of clinical trials. The India-developed DNA vaccine (ZyCoV-D) showed a good safety profile with a needle-free injection system [14,15]. Our pGX9501 DNA vaccines were administrated with in vivo electroporation, which has been proven as one of the most efficient DNA delivery methods available to date [2,16]. In the past decade, several clinical studies have shown good safety and immunogenicity of DNA vaccines delivered by electroporation [1,7,17,18]. pGX9501, as a plasmid DNA vaccine administered ID followed by electroporation, had a favorable safety and tolerability profile in the finished phase I and phase II [19] studies in the US. This is in accordance with the results obtained in our study, which showed that the DNA vaccine pGX9501 (0.5~2.0 mg) also had a good safety profile in healthy Chinese subjects. Moreover, immunization was greatly boosted without a significant increase in the frequency of TRAEs after the second dose, which is an attractive feature of our vaccines, similar to completed studies and some other DNA vaccines [1,2,5,7,14,17].

In this study, the incidence rate of solicited AEs, including injection-site and systemic solicited AEs, in the vaccine group was a little lower than that in the placebo group (88.9% vs. 100%). Moreover, the rate of solicited injection-site AEs observed for the DNA vaccine pGX9501 was slightly more than those reported for other DNA vaccines, but the rate of solicited systemic AEs was similar to those reported for other DNA vaccines [7,18]. This also conformed to the characteristics of vaccines with an electroporation pathway in delivery. All the TRAEs, solicited AEs, and non-solicited AEs did not significantly appear to increase in frequency with dose escalation. There was no observed difference in the occurrence of injection-site reactions and solicited systemic AEs between different dose groups.

In this study, the immunogenicity results of the DNA vaccine pGX9501 indicated that the humoral immune response was enhanced with increasing vaccine dosages. The geometric mean titer (GMT) of binding antibodies significantly increased across all dosage groups, and the seroconversion rate reached a peak on day 42, demonstrating a positive correlation between vaccine dosage and immune response. However, the neutralizing antibody response did not reach the desired levels; even in the high-dose 2.0 mg group, the GMT was 16.0 on day 42 and dropped to 7.6 by day 58. There is room for improvement in the vaccine-induced neutralizing antibody response, suggesting that further optimization of the vaccine’s design and immunization schedule is needed in future studies to enhance the production of neutralizing antibodies. Since the SARS-CoV-2 Beta variant started to circulating during the clinic trial, a live virus neutralization assay against the Beta variant strain (CSTR.16698.06.NPRC 2.062100001) was performed. Regarding the possibility of obtaining a higher Nab against other variants, it may happen, since our preclinic results show that the DNA vaccine also can generate higher Nab titers against WT and Delta variants.

In terms of cellular immunity, IFN-γ ELISpot analysis showed a significant increase in IFN-γ levels post-vaccination, indicating that the vaccine successfully induced cell-mediated immune responses. Combined with the humoral immunity data, this suggests that the DNA vaccine pGX9501 can effectively activate a comprehensive immune response, including cellular immunity.

Furthermore, our findings regarding differences in microbiome structure, beta diversity, alpha diversity, and bacterial species indicate baseline microbiome differences between vaccine response groups, with high-response groups showing greater microbial diversity and increased abundance of specific microbial communities. This suggests that the gut microbiota may play a potential role in modulating immune responses to DNA vaccines. It is documented that immune responses to vaccination are variable between different individuals or populations. Scientific evidence has proved that the gut microbiota is a crucial factor for modulating immune responses to vaccination. In detail, the gut microbiota metabolizes proteins and complex carbohydrates and produces an enormous number of metabolic products that can mediate crosstalk between antigens presenting in immune cells. In addition, the diversity of the gut microbiota can affect the immune response after vaccination. In this study, the high-response group exhibited higher microbial diversity, including significantly more observed microbial genes and a relatively higher Shannon diversity index compared to the lower-response groups. Further, we found that there are variations in beta diversity between the high-response and low-response groups, in which an increased abundance of *Bacteroides eggerthii*, *Ruminococcus bromii*, and *Streptococcus infantis* microbiota were found in the high-response groups. Those observed differences in microbial taxa and genes between the high- and low-response groups led to varied microbial functions, including higher metabolism in carbohydrates, amino acids, and lipids in the high-response group. All those combined factors may lead to different immune responses after vaccination with a DNA vaccine.

In conclusion, although the performance of the DNA vaccine pGX9501 in terms of neutralizing antibody levels did not meet the desired standards, the immunogenicity and safety data provided by this study, along with insights into the impact on the gut microbiome, will provide valuable experience and references for the development of future DNA vaccines. These efforts will help us optimize vaccine design, improve the quality and duration of immune responses, and further explore the regulatory role of the gut microbiome in vaccine immune responses, laying a scientific foundation for the development of more effective and safer DNA vaccines to address various public health challenges.

## Figures and Tables

**Table 1:** Demographic and baseline characteristics (mITT analysis set).

	0.5 mg (N = 12)	1.0 mg (N = 12)	2.0 mg (N = 12)	Placebo (N = 9)	Total (N = 45)
**Age (Years)**					
n	12	12	12	9	45
Mean (SD)	29.8 (11.30)	34.8 (9.92)	27.8 (6.90)	29.2 (9.31)	30.5 (9.57)
Median	26.0	33.0	26.0	24.0	27.0
Min	21	20	21	22	20
Max	54	52	40	48	54
**Age Group**					
n	12	12	12	9	45
≤40 Years	10 (83.3%)	9 (75.0%)	12 (100.0%)	7 (77.8%)	38 (84.4%)
>40 Years	2 (16.7%)	3 (25.0%)	0	2 (22.2%)	7 (15.6%)
**Sex**					
n	12	12	12	9	45
Male	8 (66.7%)	10 (83.3%)	9 (75.0%)	4 (44.4%)	31 (68.9%)
Female	4 (33.3%)	2 (16.7%)	3 (25.0%)	5 (55.6%)	14 (31.1%)
**Race**					
n	12	12	12	9	45
Asian	12 (100.0%)	12 (100.0%)	12 (100.0%)	9 (100.0%)	45 (100.0%)
Black	0	0	0	0	0
White	0	0	0	0	0
Other	0	0	0	0	0
**Chinese Nationality**					
n	12	12	12	9	45
Han	12 (100.0%)	11 (91.7%)	10 (83.3%)	9 (100.0%)	42 (93.3%)
Other	0	1 (8.3%)	2 (16.7%)	0	3 (6.7%)

## Data Availability

The data generated or analyzed during this study are included in this published article and its Supplementary Materials.

## Supplementary Materials

The following supporting information can be downloaded at: https://www.mdpi.com/article/10.3390/vaccines13060573/s1, Figure S1. Composition of health vaccinee’s gut microbiome on phylum level; Figure S2. Composition of health vaccinee’s gut microbiome on genus level and the distribution of three enterotypes based on the most abundant genus. Table S1: The binding antibody titer and seroconversion rate of pGX9501 at Days 0, 42 and 58; Table S2: The neutralizing antibody titer and serocinversion rate of pGX9501 at Days 0, 42 and 58; Table S3: The IFN-γ Response in Peptide Pools at Days 0, 42 and 58; Table S4: The group assignment of 36 participants with successful faces collection before vaccination.
