# Supplementary material for: Safety, Tolerability, and Immunogenicity of a DNA Vaccine (pGX9501) Against SARS-CoV-2 in Healthy Volunteers: A Single-Center, Randomized, Double-Blind, Placebo-Controlled, and Dose-Ranging Phase I Trial
Source: Vaccines (Basel). 2025 May 27;13(6):573. doi: 10.3390/vaccines13060573 (PMC12197376; doi:10.3390/vaccines13060573)
Supplement: Supplementary file 1 [file vaccines-13-00573-s001.zip › vaccines-3621657-supplementary.pdf]

Figures

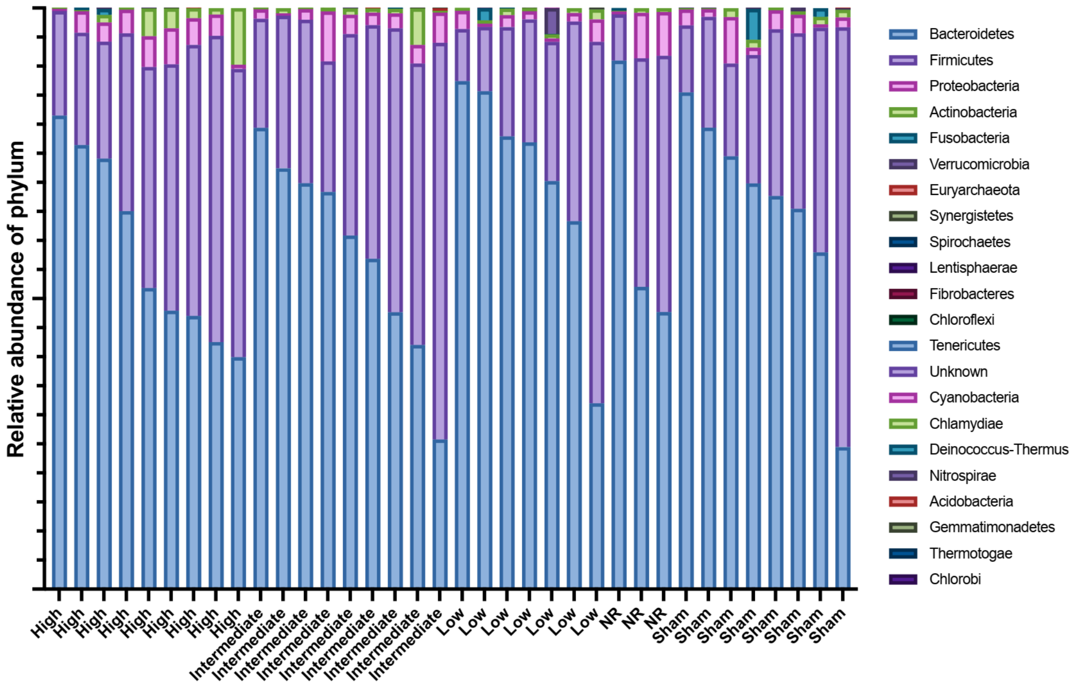

Supplement Figure S1. Composition of health vaccinee's gut microbiome on phylum level.

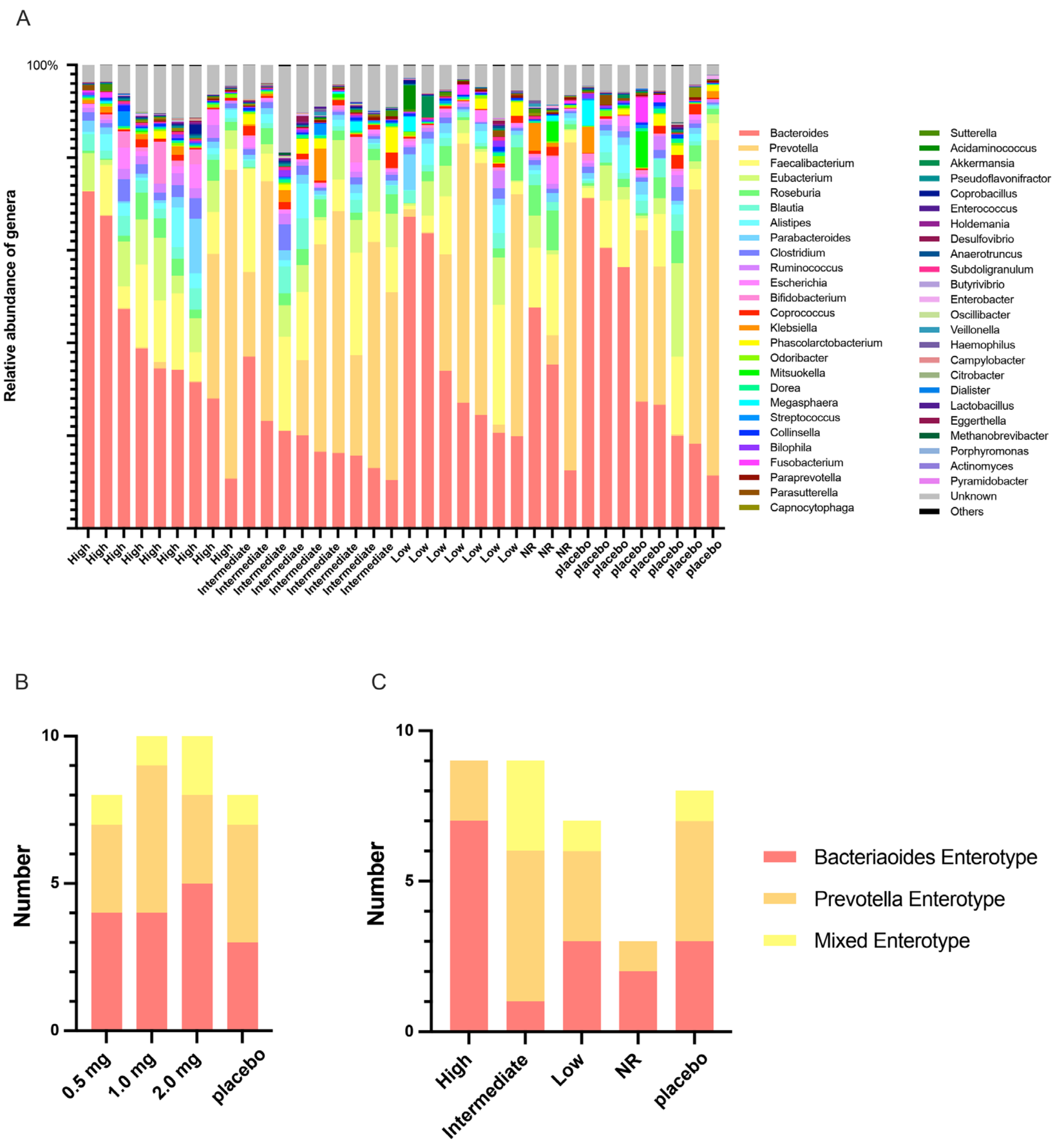

Supplement Figure S2. Composition of health vaccinee's gut microbiome on genus level and the distribution of three enterotypes based on the most abundant genus.

Tables

Supplement Table S1. The binding antibody titer and seroconversion rate of pGX9501 at Days 0, 42 and 58

IgG Titer	Pooled Placebo	0.5mg	1.0mg	2.0mg
**Day 0**				
**n**	9	12	12	12
**GMT**	200.0	200.0	200.0	200.0
**95%CI**	200.0, 200.0	200.0, 200.0	200.0, 200.0	200.0, 200.0
**Positive**	0	0	0	0
**Seroconversion Rate**	0%	0%	0%	0%
**95%CI**	0%, 0%	0%, 0%	0%, 0%	0%, 0%
**Day 42**				
**n**	9	12	12	12
**GMT**	200.0	1067.9	1695.1	4525.5
**95%CI**	200, 200	392.1, 2908	710.3, 4045.7	2329.8, 8790.4
**Positive**	0	7	9	11
**Seroconversion Rate**	0.0%	58.3%	75.0%	91.7%
**95%CI**	0%, 33.6%	27.7%, 84.8%	42.8%, 94.5%	61.5%, 99.8%
**Day 58**				
**n**	9	12	12	12
**GMT**	200.0	847.6	1067.9	2262.7
**95%CI**	200, 200	378.3, 1899.2	510.3, 2234.5	1196.9, 4277.6
**Positive**	0	7	9	11
**Seroconversion Rate**	0.0%	58.3%	75.0%	91.7%
**95%CI**	0%, 33.6%	27.7%, 84.8%	42.8%, 94.5%	61.5%, 99.8%

Supplement Table S2. The neutralizing antibody titer and seroconversion rate of pGX9501 at Days 0, 42 and 58

Nab Mean Titer	Pooled Placebo	0.5mg	1.0mg	2.0mg
**Day 0**				
**n**	9	12	12	12
**GMT**	0.5	0.5	0.5	0.5
**95%CI**	0.5, 0.5	0.5, 0.5	0.5, 0.5	0.5, 0.5
**Positive**	0	0	0	0
**Seroconversion Rate**	0%	0%	0%	0%
**95%CI**	0%, 0%	0%, 0%	0%, 0%	0%, 0%
**Day 42**				
**n**	9	12	12	12
**GMT**	0.5	3.4	5.3	16.0
**95%CI**	0.5, 0.5	1.3, 8.8	2.2, 13	7.2, 35.5
**Positive**	0	8	10	11
**Seroconversion Rate**	0.0%	66.7%	83.3%	91.7%
**95%CI**	0%, 33.6%	34.9%, 90.1%	51.6%, 97.9%	61.5%, 99.8%
**Day 58**				
**n**	9	12	12	12
**GMT**	0.5	2.0	2.7	7.6
**95%CI**	0.5, 0.5	1, 3.8	1.2, 5.7	3.2, 17.7
**Positive**	0	7	7	10
**Seroconversion Rate**	0.0%	58.3%	58.3%	83.3%
**95%CI**	0%, 33.6%	27.7%, 84.8%	27.7%, 84.8%	51.6%, 97.9%

Supplement Table S3. The IFN -γ Response in Peptide Pools at Days 0, 42 and 58

IFN -γ (Peptide Pool)	Pooled Placebo	0.5mg	1.0mg	2.0mg
**Day 0**				
**n**	9	11	12	12
**Mean**	13.89	81.67	56.39	74.17
**95%CI**	2.18, 25.6	-10.63, 173.96	-9.22, 121.99	-21.3, 169.63
**Day 42**				
**n**	9	12	12	11
**Mean**	14.7	448.06	332.79	161.11
**95%CI**	-4.26, 32.41	25.45, 870.66	-48.08, 713.63	1.48, 320.74
**Day 58**				
**n**	9	12	12	12
**Mean**	18.15	504.17	270.83	479.17
**95%CI**	-7.32, 43.61	2.12, 1006.2	-35.2, 576.86	160.76, 797.57

Supplement Table S4. The group assignment of 36 participants with successful feces collection before vaccination

Enrollment No.	Vaccination group	Immuno response at D42	Immuno response at D58	Group assignment
49	0.5 mg	High	High	High
99	1.0 mg	High	High	High
103	1.0 mg	High	High	High
122	1.0 mg	High	High	High
168	2.0 mg	High	High	High
169	2.0 mg	High	High	High
170	2.0 mg	High	High	High
175	2.0 mg	High	High	High
180	2.0 mg	High	High	High
6	0.5 mg	High	Intermediate	Intermediate
40	0.5 mg	High	Intermediate	Intermediate
91	1.0 mg	Intermediate	High	Intermediate
106	1.0 mg	High	Intermediate	Intermediate
125	1.0 mg	High	Intermediate	Intermediate
181	2.0 mg	High	Intermediate	Intermediate
52	0.5 mg	Intermediate	Intermediate	Intermediate
131	2.0 mg	Intermediate	Intermediate	Intermediate
179	2.0 mg	Intermediate	Intermediate	Intermediate
5	0.5 mg	Intermediate	Low	Low
23	0.5 mg	Low	Intermediate	Low
33	0.5 mg	Low	Intermediate	Low
59	1.0 mg	Intermediate	Low	Low
174	2.0 mg	Intermediate	Low	Low
90	1.0 mg	Low	Low	Low
173	2.0 mg	Low	Low	Low
93	1.0 mg	N/A	N/A	NR
108	1.0 mg	N/A	N/A	NR
12	0.5 mg	N/A	N/A	NR
32	placebo	N/A	N/A	placebo
56	placebo	N/A	N/A	placebo
88	placebo	N/A	N/A	placebo
100	placebo	N/A	N/A	placebo
101	placebo	N/A	N/A	placebo
172	placebo	N/A	N/A	placebo
182	placebo	N/A	N/A	placebo
186	placebo	N/A	N/A	placebo
